# Mechanical Properties of Composite Core Build-Up Materials: A Comparative Study

**DOI:** 10.3390/ma19081487

**Published:** 2026-04-08

**Authors:** Emily Mundy, Sanaya V. Engineer, Sheila Butler, Amin Rizkalla, Gildo Coelho Santos Junior, Maria Jacinta Moraes Coelho Santos

**Affiliations:** Division of Restorative Dentistry, Schulich School of Medicine and Dentistry, Western University, London, ON N6A 3K7, Canada; emundy2025@dents.uwo.ca (E.M.); sheila.butler@schulich.uwo.ca (S.B.); arizkall@uwo.ca (A.R.)

**Keywords:** core build-up materials, bulk fill composite, dual-cure resin, mechanical properties, water sorption, solubility

## Abstract

**Objective:** To determine the most suitable core build-up materials based on their mechanical and physical properties, different resin based materials were evaluated for flexural strength (FS), flexural modulus (E), modulus of resilience (R), water sorption (WS), and solubility (SO). **Materials and Methods:** Three dual-cure resins (CosmeCore DC Automix, CCC; Clearfil DC Core Plus, CCP; MultiCore Flow, CMC) and two bulk fill composites (Filtek One Bulk Fill Restorative, BFO; Filtek Bulk Fill Flowable, BFF) were tested, with Filtek Supreme Ultra (FSU) as the control. All tests followed ISO 4049. Beam specimens (25 × 2 × 2 mm, n = 12) were used to determine FS and E after 24 h storage in 37 °C deionized water, using a three-point bending test. Disc specimens (15 × 1 mm, n = 5) were used for WS and SO by measuring mass changes before and after water storage. Data were analysed using one way ANOVA and Tukey post hoc tests (*p* < 0.05). **Results:** CCC exhibited the highest FS and lowest WS. BFF showed the lowest E, while BFO exhibited the highest R. FSU demonstrated the lowest FS and R, along with the highest WS. No significant differences in SO were observed among groups. **Conclusions:** The evaluated materials showed considerable variation in mechanical and physical properties. CCC and BFO demonstrated the most favourable performance, suggesting they are the most suitable candidates for core build up procedures among the materials tested.

## 1. Introduction

Restorative dentistry has witnessed substantial advancements in materials and techniques aimed at reconstructing compromised tooth structure and ensuring long-term clinical success. Among endodontically treated or severely broken-down teeth, which are often significantly weakened due to the loss of coronal hard tissue, a core build-up restoration provides the structural foundation necessary to support a subsequent extracoronal restoration [1,2]. Resin core build-up materials have gained considerable clinical favourability due to their versatility, excellent adhesion to tooth structure, ability to be sculptured and manipulated chairside, and compatibility with contemporary adhesive systems [3,4].

Core build-up materials are typically composed of a resin matrix such as dimethacrylate (Bis-GMA) or urethane dimethacrylate (UDMA) reinforced with inorganic fillers such as glass, quartz, or silica particles [5]. These fillers serve a critical biomechanical function: they reduce the proportion of the polymeric matrix, thereby limiting water sorption and improving resistance to deformation under load. The filler content has been shown to be a key determinant of mechanical performance, with increased filler loading at a threshold of approximately 55–57% by volume associated with higher fracture toughness and flexural strength [6,7]. Additionally, these materials may contain initiators, accelerators, and pigments to control the polymerization process and achieve desired aesthetic outcomes [5].

Despite their advantages, resin core build-up materials present certain clinical challenges. Polymerization shrinkage can produce internal stresses and marginal gaps, potentially leading to microleakage, marginal discoloration, and secondary caries if not properly managed [8]. In response to these limitations, bulk-fill resin composite materials have been developed to permit the placement of increments up to 4–5 mm, thereby reducing the number of required layers and overall chairside time [9]. These materials overcome the depth-of-cure limitations of conventional composites through increased translucency, enhanced photoinitiators, and specially engineered filler systems that reduce light scattering [10]. Furthermore, some bulk-fill formulations incorporate stress-relieving monomers, such as addition-fragmentation monomers, to mitigate polymerization shrinkage stress associated with bulk placement [11].

Bulk-fill resin composites are classified as either high-viscosity (sculptable) or low-viscosity (flowable) types [12]. High-viscosity bulk-fill composites typically contain a higher filler concentration and are preferred for stress-bearing applications [13], whereas flowable bulk-fill composites exhibit comparatively lower mechanical properties and are more often used as base or liner materials [14]. In contrast, dual-cure resin core build-up materials combine the benefits of both light and self-curing polymerization mechanisms, ensuring adequate conversion in areas where light penetration may be limited such as deep cavities or regions apical to a post [15,16]. Recent studies have demonstrated that bulk-fill resins exhibit superior polymerization shrinkage and depth of cure compared to conventional resin composites and dual-cured core resins, and that at 4 mm depth, bulk-fill resin composites achieved an appropriate depth of cure, while for dual-cured core resins the depth of cure varied depending on the product [17]. Additionally, light-curing significantly improves the flexural modulus of dual-cure core build-up materials on the coronal aspect, reinforcing the clinical recommendation to light-cure these restorations following placement [18,19].

The properties of core build-up materials including flexural strength (FS), flexural modulus (E), modulus of resilience (R), water sorption (WS), and water solubility (SO) play crucial roles in determining their clinical performance and durability [1,20]. FS, a measure of a material’s resistance to bending or breaking under applied load, is a key indicator of structural integrity and the ability to withstand occlusal forces. Higher filler content is generally associated with enhanced FS, as the fillers provide reinforcement to the resin matrix and resist deformation under stress [6,7]. E reflects the stiffness of a material and influences its ability to distribute stress within the restoration; core build-up materials with an elastic modulus more closely matching that of dentin (11–20 GPa) are considered biomechanically favourable, as they reduce interfacial stress generation [1]. R quantifies a material’s capacity to absorb energy elastically before permanent deformation, which is critical for withstanding repetitive loading cycles in the oral environment [21,22]. Furthermore, research has established a significant positive correlation between flexural strength and both shear bond strength to root dentin and post retention force, underscoring the broader clinical relevance of flexural testing beyond simple mechanical characterisation [23].

In addition to mechanical properties, the interaction of core build-up materials with oral moisture is a significant clinical consideration. WS refers to the amount of water absorbed by the material over time, which exerts a plasticizing effect on the polymer matrix, reducing both flexural modulus and flexural strength [24]. Hydrolytic degradation of the dimethacrylate-based resin matrix, which involves cleavage of ester bonds and breakdown of silane coupling agents bonding fillers to the matrix, further compromises material integrity over time [25,26]. SO measures the extent to which material components dissolve in water, indicating susceptibility to degradation and loss of structural integrity, as well as potential release of residual monomers with associated biocompatibility concerns [27]. Monomers containing hydrophilic functional groups, such as Bis-GMA and TEGDMA, have been associated with elevated WS and SO values, while higher filler content and more hydrophobic matrix compositions tend to reduce these values [28,29]. Water sorption and solubility of resin-based core build-up materials have been shown to exhibit time-dependent increases, with bulk-fill composites demonstrating higher water sorption and solubility values compared to conventional composites at greater depths, attributed in part to reduced polymerization conversion with increasing distance from the light source [30]. Similarly, monomer composition has been identified as a predominant factor governing physicochemical stability, while filler characteristics such as loading, content, and distribution dictate mechanical performance [31].

Given the expanding variety of commercially available core build-up materials and the clinical importance of selecting the most appropriate option, comprehensive comparative evaluation of their mechanical and physical properties is warranted. While prior investigations have examined individual properties of some of these materials in isolation, a direct simultaneous comparison of flexural properties, modulus of resilience, and water sorption/solubility across this specific combination of dual-cure resins, bulk-fill composites, and a conventional composite control under standardised ISO 4049 conditions is lacking in the current literature. This study addresses that gap by providing a comprehensive, standardised, side-by-side evaluation to guide evidence-based material selection for core build-up procedures. Specifically, it examines the FS, E, R, WS, and SO of six materials: three dual-cure core build-up resins CosmeCore DC Automix (Cosmedent; CCC), Clearfil DC Core Plus (Kuraray; CCP), and MultiCore Flow (Ivoclar Vivadent; CMC); two bulk-fill resin composites Filtek One Bulk Fill Restorative (3M ESPE; BFO) and Filtek Bulk Fill Flowable (3M ESPE; BFF); and one conventional resin composite control Filtek Supreme Ultra (3M ESPE; FSU). The null hypothesis is that there will be no significant differences in FS, E, R, WS, or SO among the six materials tested.

## 2. Materials and Methods

A total of 102 specimens were fabricated from the following materials: three core build-up resins (CCC, CCP, and CMC), two bulk-fill resin composites (BFO and BFF), and one conventional resin composite control (FSU). Material compositions, filler contents, manufacturers, and batch numbers are presented in Table 1. All sample preparation and tests were conducted in accordance with ISO 4049 [32].

### 2.1. Flexural Strength and Flexural Modulus Testing

**Figure 1:**
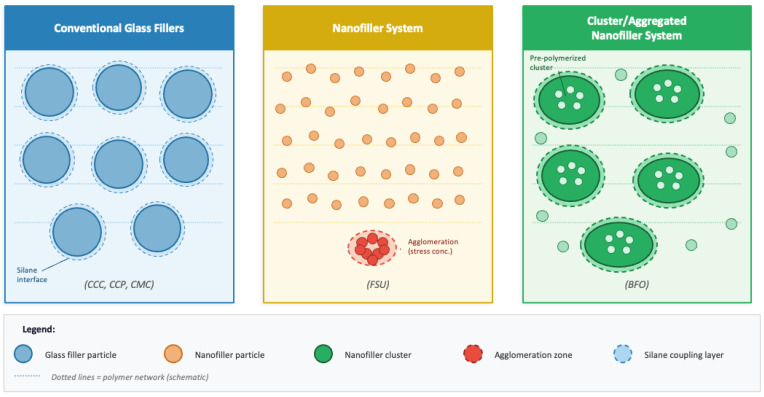
Schematic representation of filler dispersion patterns within the resin matrix for the three material categories tested: conventional glass filler composites (CCC, CCP, CMC), nanofiller composites (FSU), and cluster/aggregated nanofiller composites (BFO). The schematic highlights the silane coupling interface, nanofiller agglomeration zones, and pre-polymerized cluster architecture.

To assess FS and E, beam-shaped specimens (25 mm × 2 mm × 2 mm, n = 12 per group) were prepared by incrementally inserting uncured resin into a metal mould atop a clear glass cover slip (Micro Slides, Gold Seal) using a hand instrument to minimise air incorporation. For automix dual-cure materials (CCC), a dispensing tip was used to deliver the resin directly into the mould, further reducing the risk of void formation. The mould was slightly overfilled, and a Mylar strip and a second glass cover slip were then firmly pressed over the open face under clamp pressure to displace excess material and ensure complete, bubble-free filling. Specimens were subsequently light-cured for 20 s per segment using an LED curing unit (Bluephase G4, Ivoclar Vivadent AG, Schaan, Liechtenstein; 1200 mW/cm^2^) in approximately 6 mm overlapping sections, first along the top surface and then along the bottom surface after flipping the assembly. For dual-cure materials, the self-curing component was allowed to proceed for an additional 5 min after light exposure before demoulding, in accordance with manufacturers’ recommendations. Irradiance output was verified using the curing unit’s built-in radiometer prior to the preparation of each group. All specimens were inspected under backlight for flaws; those exhibiting cracks, porosity, or material deficiency were discarded. Acceptable specimens were polished on all six sides with 600-grit silicon carbide paper, dimensionally measured using a digital calliper (Starrett, 0.001 mm accuracy), and stored individually in deionized water at 37 °C for 24 h prior to testing. The structural differences in filler dispersion among the three material categories evaluated in this study are schematically illustrated in Figure 1.

**Figure 2:**
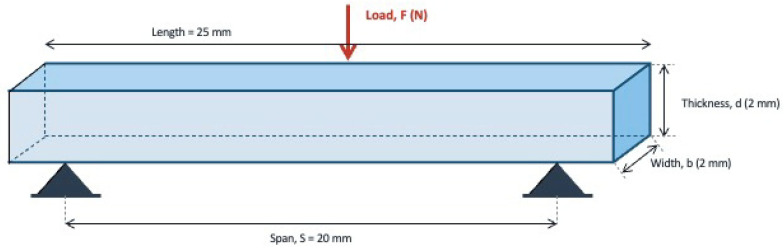
Schematic diagram of the support beam and sample.

Flexural strength was evaluated using a three-point bending test on a universal testing machine (411 Instron Universal Testing Machine, Instron Corp., Canton, MA, USA; Test Works 3.3 Software) at a cross-head speed of 0.75 mm/min, as per Figure 2. FS was calculated using the formula:

*FS* = 3*SF_max_*/2*bd*^2^

where *F_max_* is the load at fracture (N), S is the support span (mm), b is the specimen width (mm), and d is the specimen thickness (mm). E was calculated from the load-deflection curves as:

*E* = *S*^3^*m*/4*bd*^3^

where m is the slope of the initial linear portion of the load/deflection curve (N/mm). R was subsequently derived as:

*R* = (*FS*)^2^/2*E*

### 2.2. Water Sorption and Solubility Testing

To measure WS and SO, disc-shaped specimens (15 mm diameter × 1 mm thickness, n = 5 per group) were prepared using a similar insertion and curing protocol. Specimen peripheries were polished with 600-grit silicon carbide paper. The specimens were desiccated at 37 °C for 22 h, followed by desiccation at 23 °C for 2 h, then weighed to an accuracy of 0.1 mg using a precision scale (AB204-S analytical balance, Mettler Toledo, Greifensee, Switzerland). This cycle was repeated until a constant mass (m_1_) was achieved, defined as mass loss of no more than 0.1 mg over a 24 h period. Specimen thickness was measured with a digital calliper and volume (V) was calculated.

Specimens were subsequently submerged in deionized water at 37 °C for 7 days, maintained with a minimum spacing of 3 mm between specimens. Specimens were then removed, blotted dry with blotting paper, waved in air for 10 s to remove excess surface moisture, and weighed to obtain m_2_. Finally, specimens were re-desiccated using the same protocol to obtain a final constant mass (m_3_). WS and SO were calculated as:

*WS* (µg/mm^3^) = (*m*_2_ − *m*_3_)/*V*

*SO* (µg/mm^3^) = (*m*_1_ − *m*_3_)/*V*

### 2.3. Statistical Analysis

One-way ANOVA and Tukey’s post hoc tests were used to compare FS, E, R, WS, and SO across all material groups. The significance level was set at α = 0.05. Statistical computations were performed using GraphPad Prism version 9 (GraphPad Software, San Diego, CA, USA).

## 3. Results

The mean values and standard deviations for FS, E, and R are presented in Table 2. CCC demonstrated the highest FS among all materials tested [137.77 (11.34) MPa], while FSU exhibited the lowest [100.17 (19.91) MPa]. BFF displayed the lowest E [4.69 (0.21) GPa], whereas FSU demonstrated the highest E [8.91 (0.82) GPa], though this result did not significantly differ from BFO (*p* > 0.05). Regarding R, BFF achieved the highest value [1.28 (0.13) MPa] and FSU the lowest [0.62 (0.20) MPa]. Notably, CCP and CMC showed no statistically significant differences across all three flexural parameters (*p* > 0.05).

Mean values and standard deviations for WS and SO are presented in Table 3. FSU exhibited the highest WS [28.76 (3.49) µg/mm^3^], while CCC demonstrated the lowest [14.92 (0.51) µg/mm^3^], suggesting that CCC may be the most dimensionally stable material under oral conditions, while FSU may be the least stable. No significant differences in SO were observed across all six material groups (*p* > 0.05).

## 4. Discussion

For this investigation, the three-point bending test, the gold standard for flexural testing of resin-based dental materials, was used to evaluate FS, E, and R, while standardised water immersion protocols were employed to generate WS and SO data [33]. Based on the results obtained, CCC and BFO demonstrated optimal properties for use as core build-up materials, exhibiting comparatively high values for FS, E, and R alongside low WS and SO values. These findings suggest that both materials offer robust mechanical properties while maintaining good stability in the oral environment, characteristics that are critical for long-term core build-up success. Based on this data, the null hypothesis, which stated no significant differences in the mechanical and physical properties of the six tested materials, was rejected.

FS is a critical parameter reflecting a material’s resistance to bending or breaking under load and is positively correlated with shear bond strength to root dentin and post retention force, underscoring its broader clinical relevance [23]. CCC exhibited the highest FS among the tested materials. This superior performance may be attributed in part to its filler composition and dual-cure polymerization mechanism [15,16]. Although CCC does not present the highest filler loading among the tested materials, its filler system comprises larger conventional glass particles that provide more effective stress transfer and matrix reinforcement under flexural loading compared to nanosized fillers [6,7]. Core build-up composites with elevated inorganic filler volumes provide greater reinforcement to the resin matrix, thereby enhancing resistance to deformation under stress [6,7]. Furthermore, the dual-cure mechanism employed by CCC ensures more complete polymerization in areas inaccessible to light alone, resulting in improved mechanical properties throughout the restoration [34]. Consistent with these findings, a comparative in vitro investigation of four core build-up materials reported that a dual-cure composite reinforced with zirconia particles demonstrated the highest flexural strength and fracture toughness among all tested materials, recommending dual-cure composites as the material of choice for core build-up procedures [35].

BFO demonstrated the next highest FS following CCC. In agreement with previous research, BFO and CMC showed similar FS values, within one standard deviation of each other [36]. Although some studies have reported higher FS values for BFF compared to BFO [37], the current investigation found these materials to be broadly comparable under flexural stresses, with BFO reporting marginally higher FS values. This may be attributed to BFO’s higher inorganic filler content and lower resin matrix proportion compared to the flowable BFF, which have been associated with improved mechanical properties [38]. Moreover, BFO’s sculptable consistency may confer greater resistance to deformation under increasing load compared to the flowable BFF [38]. BFO incorporates a nanofiller system specifically engineered to promote strength and wear resistance for use in stress-bearing areas, further contributing to its superior mechanical performance [39].

FSU demonstrated the lowest FS among the materials tested. As a nanofilled composite, FSU employs nanosized particles of silica and zirconia primarily to achieve superior aesthetics and polishability [40]. However, despite presenting the highest filler loading among all materials tested (78.5 wt%, 59.5 vol%), FSU’s inferior FS performance suggests that filler content alone does not determine mechanical outcomes. The nanosized morphology of FSU’s silica and zirconia particles, while optimising optical properties and polishability, may provide less effective stress transfer and matrix reinforcement under flexural loading compared to the larger conventional glass filler particles found in dedicated core build-up materials [6,7,40]. From a polymer physics perspective, the dramatically higher surface-area-to-volume ratio of nanoparticles challenges the uniformity and completeness of silane surface treatment. Incomplete or heterogeneous silane coupling at the filler-matrix interface weakens interfacial adhesion, facilitating debonding and crack propagation under flexural stress [6,7]. Additionally, nanoparticles at high loading levels exhibit a thermodynamic tendency toward agglomeration, forming local particle clusters that act as stress concentration points within the matrix and paradoxically reduce FS despite the elevated overall filler content. These structural differences between material filler systems are schematically illustrated in Figure 1. Furthermore, FSU was designed as a direct restorative material with aesthetics and handling characteristics at the forefront of its design, a contrast to core build-up materials, which prioritise strength over aesthetics [37].

The highest E value, reflecting material stiffness, was obtained from FSU, though this result was not significantly different from BFO. FSU’s elevated E may be explained by its highly cross-linked Bis-GMA resin matrix, which provides rigidity and resistance to deformation, but also renders the material more brittle and less able to plastically deform under stress [41]. BFF performed most poorly in terms of E. As a flowable composite, BFF was designed with a lower filler content compared to the sculptable BFO, resulting in decreased stiffness and greater flowability [37,42]. These results suggest that while BFF may be beneficial as a stress-absorbing base beneath more rigid restorative materials, it may not be suitable in areas of high occlusal loading compared to higher-viscosity alternatives. This finding aligns with the established classification of bulk-fill composites, whereby flowable bulk-fill types, with their comparatively lower mechanical properties, require an additional 2 mm occlusal capping layer of a high-viscosity material when used in posterior restorations [13,14].

R, quantifying a material’s capacity to absorb energy elastically before permanent deformation, showed a trend similar to FS, with CCC and BFO outperforming the other materials and FSU demonstrating the least R. This can be explained by the mechanical characteristics discussed above: CCC and BFO incorporate filler systems and matrix compositions specifically engineered for stress-bearing applications [6,11], while FSU’s nanofiller technology, although present at high loading levels, is optimised for optical performance and polishability rather than flexural reinforcement [40]. Despite FSU’s high E, its comparatively lower FS and R suggest it may not be the optimal choice for core build-up applications. Unlike posterior restorations, where occlusal forces are distributed across a broader surface supported by remaining tooth structure, a core build-up operates as a stress-transmitting intermediary between the overlying crown and the root, often in the context of reduced dentinal walls. This configuration subjects the core material to more complex, concentrated, and multidirectional forces, conditions that favour materials with higher FS and R to resist fracture and absorb energy under functional loading.

WS and SO are important indicators of a material’s long-term stability in the oral environment. Water sorption in resin composites predominantly occurs through diffusion into the polymer matrix, exerting a plasticizing effect that reduces both flexural modulus and flexural strength over time [24]. At the molecular level, monomers such as Bis-GMA and TEGDMA contain hydroxyl groups and flexible ether linkages that promote hydrogen bonding with water molecules, increasing water uptake at the polymer network level. TEGDMA’s relatively low molecular weight and bifunctional structure also increase free volume and reduce cross-link density within the polymer network when present in excess, creating additional diffusion pathways for water. In contrast, UDMA and Bis-EMA possess fewer polar pendant groups and contribute to a more densely cross-linked, hydrophobic network that restricts water diffusion. Hydrolytic degradation of ester bonds within the dimethacrylate matrix, as well as breakdown of the silane coupling agents bonding fillers to the resin, further compromises material integrity with prolonged water exposure [25,26]. High WS and SO values can therefore compromise restoration longevity through dimensional changes, degradation of mechanical properties, and leaching of residual monomers with potential biocompatibility implications [27]. Water sorption predominantly occurs in the resin matrix, and an inverse relationship between filler content and water sorption has been demonstrated, such that as filler content increases, the relative proportion of the polymer matrix decreases, leading to lower WS and SO values [30,31]. This mechanism is consistent with the composition-dependent behaviour observed across the materials tested in the present study. It should be noted that flexural testing in this study was performed after 24 h of water storage per ISO 4049, whereas WS and SO measurements involved 7 days of immersion as a separate protocol. These protocols were conducted independently, and therefore the present data do not permit direct conclusions about the magnitude of mechanical property changes caused by extended water exposure. The established plasticizing and degradation effects are cited here as well-documented contextual background [24,25] rather than as direct conclusions from the current dataset.

In the present study, CCC demonstrated the lowest WS, while FSU exhibited the highest. These findings align with the established relationship between hydrophilic monomer composition and water uptake behaviour: materials containing high proportions of Bis-GMA and TEGDMA as found in FSU demonstrate elevated WS due to the polar functional groups within these monomers, which facilitate water diffusion into the polymer network [28]. In contrast, CCC, with its more hydrophobic matrix composition based on UDMA and Bis-EMA monomers, demonstrated lower WS values, as these monomers present fewer polar functional groups available for water diffusion compared to the Bis-GMA and TEGDMA-rich matrix of FSU [28]. Specifically, the hydroxyl groups on the Bis-GMA backbone and the ether linkages of TEGDMA promote hydrogen bonding with water molecules, while TEGDMA’s lower molecular weight increases network free volume and reduces cross-link density, facilitating water diffusion. UDMA and Bis-EMA, by contrast, lack these strongly polar groups and their bulkier molecular structure contributes to a more densely cross-linked network with reduced water permeability [28]. These findings are further supported by investigations demonstrating that resin-based composites with optimised monomer composition and high filler content exhibit superior mechanical properties alongside lower WS and SO [43]. CCP, CMC, BFO, and BFF all performed comparably for WS and SO. No significant differences in SO were observed across all groups, with values indicating minimal to no material dissolution, consistent with the findings of Zankuli et al. [20], who reported SO values of similar magnitude for comparable materials using ISO 4049. Furthermore, resin-based core build-up materials have demonstrated significantly lower sorption and solubility values compared to glass ionomer cement-based alternatives across varying pH media, underscoring their superior dimensional stability in the chemically dynamic oral environment [44]. The superior overall performance of CCC, particularly its combination of high FS and low WS, aligns with the findings of Bitter et al. [45].

Taken together, the present findings reveal meaningful differences in FS, E, R, and WS among the six tested materials, which clinicians should consider on a case-by-case basis when selecting the most appropriate restorative material for core build-up procedures. CCC and BFO emerge as the most mechanically suitable candidates based on the properties evaluated in this study. It should be acknowledged as a limitation of this investigation that only flexural-based mechanical parameters and water-related physical properties were assessed, as prescribed by ISO 4049. Compressive strength, surface hardness, and wear resistance are also clinically relevant for restorative materials and were not evaluated in the present study; future work should include these parameters to provide a more complete mechanical profile. Further research is also warranted to explore the clinical performance and biocompatibility of these materials in vivo, incorporating additional parameters such as marginal adaptation, bond strength to dentin, and fracture resistance of restored teeth. Some attempts to compile clinical data on conventional and bulk-fill resin composites have been made; however, systematic reviews have thus far been unable to identify significant differences in clinical outcomes between these material categories [46].

## 5. Conclusions

Within the limitations of this in vitro study, the results demonstrate that the tested materials differed significantly in their mechanical and physical properties. CCC and BFO exhibited the highest FS and R combined with low WS values, identifying them as the most mechanically suitable candidates for core build-up applications among the materials evaluated. FSU demonstrated the highest E and WS alongside the lowest FS and R, a profile inconsistent with the demands of a stress-bearing core restoration, despite its high filler content. BFF exhibited the lowest E among all groups, consistent with its flowable formulation and lower filler loading. No significant differences in SO were observed across any of the six materials tested (*p* > 0.05). Future studies should extend this characterisation to include compressive strength, surface hardness, and wear resistance, and should incorporate ageing protocols such as thermocycling and long-term immersion in artificial saliva, with subsequent validation through clinical trials to confirm the in vivo performance of the recommended materials.

## Figures and Tables

**Table 1:** Composition of the materials used in this study.

Material	Manufacturer	Composition (Monomer System/Filler System)	Filler Content
CosmeCore DC Automix—CCC	Cosmedent, Chicago, IL, USA	Matrix: UDMA (~40%), Bis-EMA (~25%), Bis-GMA (~20%), tricyclodecanedimethanol diacrylate, 1,4-butanediol dimethacrylate. Filler: silanated barium aluminosilicate glass (1–3 μm), fumed silicon dioxide (nanosilica). 69 wt%/48 vol%.	69 wt%, 48 vol%
Clearfil DC Core Plus—CCP	Kuraray, New York, NY, USA	Matrix: Bis-GMA (~55%), TEGDMA and aromatic/aliphatic dimethacrylates. Filler: silanated barium aluminosilicate glass particles (1–2 μm), colloidal silica, alumina. 74 wt%/52 vol%.	74 wt%, 52 vol%
MultiCore Flow—CMC	Ivoclar Vivadent, Schaan, Liechtenstein	Matrix: Bis-GMA (~40%), UDMA (~35%), TEGDMA (~10%). Filler: silanated barium aluminosilicate glass (0.7 μm avg), ytterbium trifluoride (radiopacifier), fumed silicon dioxide. 68 wt%/47 vol%.	68 wt%, 47 vol%
Filtek One Bulk Fill Restorative—BFO	3M ESPE, St. Paul, MN, USA	Matrix: AUDMA (addition-fragmentation monomer for shrinkage stress relief), DDDMA, UDMA, 1,12-dodecanediol dimethacrylate. Filler: pre-polymerized zirconia/silica cluster fillers (0.6–10 μm) and silica nanofillers (20 nm). 76.5 wt%/58.4 vol%.	76.5 wt%, 58.4 vol%
Filtek Bulk Fill Flowable—BFF	3M ESPE, St. Paul, MN, USA	Matrix: AUDMA, DDDMA, procrylat resin (proprietary low-shrinkage monomer). Filler: zirconia/silica cluster fillers and silica nanofillers (20 nm). 64.5 wt%/42.5 vol%.	64.5 wt%, 42.5 vol%
Filtek Supreme Ultra—FSU (control)	3M ESPE, St. Paul, MN, USA	Matrix: Bis-GMA (~55%), UDMA (~15%), TEGDMA (~10%), Bis-EMA (~20%). Filler: aggregated zirconia/silica nanoclusters (0.6–1.4 μm) and discrete silica nanofillers (20 nm). 78.5 wt%/59.5 vol%.	78.5 wt%, 59.5 vol%

**Table 2:** Means and standard deviations of mechanical properties.

Material	Flexural Strength—FS (MPa)	Flexural Modulus—E (GPa)	Modulus of Resilience—R (MPa)
CosmeCore DC Automix—CCC	137.77 (11.34) ^c^	7.82 (0.31) ^a,c^	1.22 (0.20) ^c,d^
Clearfil DC Core Plus—CCP	122.12 (12.65) ^a,b^	7.87 (0.40) ^a,b^	0.96 (0.20) ^a,b^
MultiCore Flow—CMC	111.50 (11.38) ^b,e,f^	7.89 (0.26) ^b,c^	0.80 (0.17) ^b,f^
Filtek One Bulk Fill Restorative—BFO	134.57 (14.85) ^c^	8.53 (0.58) ^d^	1.09 (0.21) ^a,d,e^
Filtek Bulk Fill Flowable—BFF	109.15 (4.61) ^a,d,e^	4.69 (0.21) *	1.28 (0.13) ^c,e^
Filtek Supreme Ultra—FSU	100.17 (19.91) ^d,f^	8.91 (0.82) ^d^	0.62 (0.20) ^f^

Same superscript letters within each column indicate no significant difference between core materials. * Significantly different from all other groups (*p* < 0.05, Tukey).

**Table 3:** Means and standard deviations of physical properties.

Material	Water Sorption—WS (µg/mm^3^)	Water Solubility—SO (µg/mm^3^)
CosmeCore DC Automix—CCC	14.92 (0.51) *	−0.45 (0.63) ^a^
Clearfil DC Core Plus—CCP	19.15 (0.90) ^a,b,c^	−0.09 (0.92) ^a^
MultiCore Flow—CMC	20.82 (1.51) ^c,e,f^	−1.18 (1.14) ^a^
Filtek One Bulk Fill Restorative—BFO	20.97 (1.70) ^b,d,f^	−0.30 (1.78) ^a^
Filtek Bulk Fill Flowable—BFF	18.30 (1.95) ^a,d,e^	−0.84 (0.95) ^a^
Filtek Supreme Ultra—FSU	28.76 (3.49) *	−0.79 (1.68) ^a^

Same superscript letters within each column indicate no significant difference between core materials. * Significantly different from all other groups (*p* < 0.05, Tukey).

## Data Availability

The original contributions presented in this study are included in the article. Further inquiries can be directed to the corresponding authors.
